# Chromosome analysis and the occurrence of B chromosomes in fish parasite *Acanthocephalus anguillae* (Palaeacanthocephala: Echinorhynchida)[Fn FN1]

**DOI:** 10.1051/parasite/2023045

**Published:** 2023-10-23

**Authors:** Martina Orosová, Anna Marková, Magda Zrzavá, František Marec, Mikuláš Oros

**Affiliations:** 1 Institute of Parasitology, Slovak Academy of Sciences Hlinkova 3 040 01 Košice Slovakia; 2 Biology Centre of the Czech Academy of Sciences, Institute of Entomology Branišovská 31 370 05 České Budějovice Czech Republic; 3 Department of Zoology, Faculty of Natural Sciences, Comenius University Ilkovičova 6 842 15 Bratislava Slovakia; 4 Faculty of Science, University of South Bohemia Branišovská 1760 370 05 České Budějovice Czech Republic

**Keywords:** Karyotype, Fluorescence *in situ* hybridization, 18S rDNA, H3 histone, B chromosomes, PCB pollution

## Abstract

The cytogenetics of Acanthocephala is a neglected area in the study of this group of endoparasites. Chromosome number and/or karyotypes are known for only 12 of the 1,270 described species, and molecular cytogenetic data are limited to rDNA mapping in two species. The standard karyological technique and mapping of 18S rRNA and H3 histone genes on the chromosomes of *Acanthocephalus anguillae* individuals from three populations, one of which originated from the unfavorable environmental conditions of the Zemplínska Šírava reservoir in eastern Slovakia, were applied for the first time. All specimens had 2*n* = 7/8 (male/female); *n* = 1m + 1m-sm + 1a + 1a (X). Fluorescence *in situ* hybridization (FISH) revealed three loci of 18S rDNA on two autosomes and dispersion of H3 histone genes on all autosomes and the X chromosome. In addition to the standard A chromosome set, 34% of specimens from Zemplínska Šírava possessed a small acrocentric B chromosome, which was always found to be univalent, with no pairing observed between the B chromosome and the A complement. The B chromosome had a small amount of heterochromatin in the centromeric and telomeric regions of the chromosomal arms and showed two clusters of H3 genes. It is well known that an environment permanently polluted with chemicals leads to an increased incidence of chromosomal rearrangements. As a possible scenario for the B chromosome origin, we propose chromosomal breaks due to the mutagenic effect of pollutants in the aquatic environment. The results are discussed in comparison with previous chromosome data from Echinorhynchida species.

## Abstract (fr)

La cytogénétique des Acanthocephala est un domaine négligé dans l’étude de ce groupe d’endoparasites. Le nombre de chromosomes et/ou les caryotypes ne sont connus que pour 12 des 1270 espèces décrites, et les données cytogénétiques moléculaires se limitent à la cartographie de l’ADNr chez deux espèces. La technique caryologique standard et la cartographie des gènes de l’ARNr 18S et de l’histone H3 ont été appliquées pour la première fois sur les chromosomes d’individus d’*Acanthocephalus anguillae* provenant de trois populations, dont l’une dans les conditions environnementales défavorables du réservoir de Zemplínska Šírava dans l’est de la Slovaquie. Tous les spécimens avaient 2*n* = 7/8 (mâle/femelle); *n* = 1m + 1m-sm + 1a + 1a (X). La technique FISH a révélé trois locus d’ADNr 18S sur deux autosomes et une dispersion des gènes de l’histone H3 sur tous les autosomes et sur le chromosome X. En plus de l’ensemble standard de chromosomes A, 34 % des spécimens de Zemplínska Šírava possédaient un petit chromosome B acrocentrique, qui s’est toujours révélé univalent, sans aucun appariement observé entre le chromosome B et le complément A. Le chromosome B avait une petite quantité d’hétérochromatine dans les régions centromériques et télomériques des bras chromosomiques et présentait deux groupes de gènes H3. Il est bien connu qu’un environnement pollué en permanence par des produits chimiques entraîne une incidence accrue de réarrangements chromosomiques. Comme scénario possible pour l’origine du chromosome B, nous proposons des cassures chromosomiques dues à l’effet mutagène des polluants du milieu aquatique. Les résultats sont discutés en comparaison avec les données chromosomiques précédentes des espèces d’Echinorhynchida.

## Introduction

Acanthocephala are a small group of gonochoric intestinal endoparasites in vertebrates with worldwide distribution. The phylum includes about 1,270 extant species with diverse life cycles involving arthropods as intermediate hosts and vertebrates as definitive hosts [54]. They are classified into four classes based on morphological characteristics [1, 34], but the validity of the fourth class, Polyacanthocephala, is being questioned based on recent molecular data [82]. Currently, despite the growing number of molecular phylogenies and the inclusion of new molecular markers in combination with morphological data, phylogenetic relationships at the family and genus levels remain unresolved and many questions remain unanswered [22, 23, 28]. Chromosomes represent important morphological units of the genome, and their characterization can be useful in phylogenetic reconstructions [16] and in understanding evolutionary patterns in organisms (e.g., [60, 71, 78, 79]). In addition, karyotypes can easily be used to reinforce proposed taxonomic changes. There are some good examples in other parasite groups such as trematodes [50, 65] and cestodes, e.g. the split of the genus *Pseudoglaridacris* from the original genus *Glaridacris* (different 2*n*) [46] or the proposed taxonomic assignment based on the obvious differences in the gross chromosome morphology of *Schistocephalus solidus* and *Ligula intestinalis* compared to other studied representatives of their families, which was discussed a decade earlier than it was accepted [49]. However, very little is known about the chromosome architecture of acanthocephalans. To date, only 15 studies have addressed this topic, with 12 species examined. Most of what we know about the “chromosome evolution” of Acanthocephala comes from classical cytogenetics. Our knowledge is limited almost exclusively to descriptions of diploid chromosome number, in some cases with chromosome morphology, and the sex chromosome system [3, 5, 6, 19, 20, 24, 29, 30, 41, 42, 47, 61, 62, 68, 76]. Some of these data were obtained more than 60 years ago from histological sections only and need to be revised. Molecular cytogenetic approaches using fluorescence *in situ* hybridization (FISH) have opened up the possibility to localize specific DNA sequences or DNA fragments on chromosomes and to identify chromosomal regions and individual chromosomes, providing detailed information on the structure of genomes and contributing to a deeper understanding of chromosome evolution [9, 37]. Among the other parasite groups where this technique has been used to analyze the chromosomal location of various markers suitable for comparative cytogenetic studies, trematodes have been the best studied [26, 27, 58, 81], followed by several cestode species (see Ref. [46]) and monogeneans [14]. However, in Acanthocephala, FISH was only applied to *Pomphorhynchus* spp. where the species-specific location of rDNA loci confirmed the validity of the two sibling species, *P. laevis* and *P. tereticollis* [3]. Using FISH, the specific telomeric repeat motifs were assigned to the telomeres (chromosome ends) of many invertebrate and vertebrate species [73]. For example, the most widespread telomeric repeat is the hexanucleotide TTAGGG motif, which is considered to be the ancestral telomeric DNA sequence for all metazoans, including the Platyhelminthes (Cestoda, Monogenea, and Trematoda) [4, 45]. However, FISH with telomeric probes, confirmed by Southern hybridization results, failed to identify the composition of chromosome ends in *Pomphorhynchus* spp. [4], suggesting either an as yet unknown telomeric motif or loss of telomeric repeats and replacement by other mechanisms of telomere maintenance.

In this study, we examined the karyotype characteristics of *Acanthocephalus anguillae* (Müller, 1780) from three populations from Slovakia, originating from sites with varying degrees of environmental pollution. *Acanthocephalus anguillae* is a common intestinal parasite of freshwater, brackish water and marine fish and the type species of the genus *Acanthocephalus* (Koelreuther, 1771). It belongs to the family Echinorhynchidae, for which published karyological data are available to date for only four of 127 valid taxa in the family (Supplementary Table S1). However, no cytogenetic study has yet been performed for *A. anguillae*. Our study was primarily aimed at determining the basic karyotype and discovering useful markers for individual chromosomes by using different staining methods and mapping multigene families (18S rDNA and H3 histone genes), which will allow future comparative studies. Since one of our sampling sites, the Zemplínska Šírava reservoir is considered one of the most contaminated with polychlorinated biphenyls (PCBs) in Europe due to long-term pollution from a nearby chemical plant [67], we also investigated whether there is a possible link between the polluted environment and chromosomal differences.

## Materials and methods

### Ethics

The chub (*Squalius cephalus*) specimens were caught by electrofishing and fishing rod at three sites in March, April, and September 2022 under a permit issued by the Ministry of Environment of the Slovak Republic (No. 47/2022). The animal study was reviewed and approved by the Ethics Committee of the Institute of Parasitology of the Slovak Academy of Sciences (Hlinkova 3, Košice, 04001, Slovakia), which also approved the implementation of the project under approval No. 1/2020/PaU. All methods used in this study were carried out in accordance with the relevant guidelines and regulations (Decree of the Ministry of the Slovak Republic No. 381/2018 Coll. and Act No. 216/2018 Coll. about fishing), and we confirm that all methods are reported in accordance with the ARRIVE guidelines [52].

### Study area and fish

The sites studied differed in the degree of environmental contamination. The first two sites, the Hnilec River (HN) (48°52′16.0″ N 20°21′52.1″ E) and the Olšava River (OL) (48°41′43.8″ N 21°24′47.7″ E), are considered clean, while the third, the Zemplínska Šírava (ZŠ) reservoir (48°47′09.0″ N 21°57′20.5″ E), is heavily polluted with PCBs [67]. A total of 151 chub (*Squalius cephalus*) were caught: 77 specimens from ZŠ, 34 specimens from HN and 40 specimens from OL. A total of 51 worms of *Acanthocephalus anguillae* were collected from fish – 36 worms from ZŠ, nine worms from HN and eight worms from OL. The prevalence and intensity of *A. anguillae* infection were 12.99% and 3.6 (1–6) in ZŠ, 18.92% and 1.29 (1–4) in HN, and 15% and 1.33 (1–2) in OL. Fish were either dissected on site or transported to the laboratory and examined the same or next day after collection. Individual parasites were rinsed in 0.9% saline immediately after isolation from the intestine of the fish host and identified microscopically by counting the hooks and hook rows and by the shape of the proboscis hook roots [38]. We found a proboscis with 10 longitudinal rows of hooks of 5–7 in each. At the same time, the isolated worms were fixed in 100% ethanol for molecular biology, and three selected specimens were reconfirmed by molecular methods (i.e., sequencing of the 18S rDNA fragment; the sequence was deposited in GenBank under accession number OR518297).

### Karyotype analysis

For karyological analysis, whole live animals were placed in a 0.025% colchicine solution for 1 h at room temperature (RT). Subsequently, 0.075 M KCl was used for hypotonic treatment. Whole intact females were treated for 4–5 h; in males, the testes were isolated and only these were incubated for 20 min at RT. Fixation was performed in two changes (30 min and 15 min) in a freshly mixed modified Carnoy’s fixative (methanol/acetic acid = 3:1) and stored at –20 °C until further use. Fragments of testes or ovarian balls were macerated in 60% acetic acid and chromosome slides were prepared using the “hot plate” spreading technique [44]. The slides were stained with 5% Giemsa solution (pH 6.8) for 30 min and rinsed twice with distilled water. Chromosome lengths were determined from digital images (taken at 100 × magnification) of 10 well-spread mitotic metaphases in males from all three sites, because the mitotically dividing oogonial metaphases were not good enough for measurement. Absolute length, relative length, and centromeric index were calculated as described in detail previously [46]. The mean and standard deviation of the length of individual chromosome pairs and their arms were calculated using Microsoft Excel. Karyotypes were organized by placing chromosome pairs in order of decreasing size. Chromosomes were classified as metacentric, submetacentric, and acrocentric according to the revised and simplified nomenclature of Levan *et al.* [36], which is based on the four-type system [17] and facilitates the definition of chromosomes and comparison of basic chromosome morphology. The comparison of the relative lengths of the corresponding chromosomes was tested for multiple dependent variables with a nonparametric Friedman’s ANOVA using Excel (Microsoft Office 2007) and the STATISTICA v.12.0 software package (StatSoft, Inc. 2013).

### Chromomycin A_3_ (CMA_3_) and DAPI staining

We performed two-color staining of chromosomes with the CMA_3_ and DAPI dyes to identify C + G-rich and A + T-rich regions on chromosomes, respectively; either sequentially or in separate experiments [56]. A stock solution of CMA_3_ (1 mg/mL) was prepared by dissolving in milliQ water for five days at 4 °C in the dark. The entire staining procedure took place in the dark. Slides, previously treated with an ethanol series (70, 80, and 100%, 1 min each), were incubated for 10 min in the working solution (stock solution of CMA_3_: McIlvaine’s buffer containing MgCl_2_; 1:1; pH = 7) at RT. Then, the slides were either stained with CMA_3_/Methyl Green for 20 min in the wet chamber or by sequential CMA_3_/DAPI banding with CMA_3_ for 15 min, followed by washing steps (McIlvaine’s buffer) and DAPI staining (0.5 μg/mL in PBS pH = 7.3, containing 1% Triton X-100) for 15 min. Finally, the edges of the slides were sealed with nail polish and the slides were stored overnight at 4 °C in the dark.

### DNA extraction, PCR amplification and DNA probe preparation and labeling

Total genomic DNA (gDNA) was extracted by the modified cetyltrimethylammonium bromide (CTAB) method [25]. The extracted gDNA served as a template for generating an 18S rDNA probe by PCR using a pair of specific primers Acant18SF (5′–AGATTAAGCCATGCATGCGTAAG–3′) and Acant18SR (5′–TGATCCTTCTGCAGGTTCACCTAC–3′) [53] under the following conditions: initial denaturation step at 95 °C for 3 min, followed by 30 cycles of denaturation at 94 °C for 1 min, annealing at 60 °C for 30 s, and extension at 72 °C for 90 s, and a final extension at 72 °C for 10 min. The 18S rDNA fragment was purified by the Wizard SV Gel and PCR Clean-Up System (Promega, Madison, WI, USA) and labeled with either biotin-16-dUTP or digoxigenin-11-dUTP (both Roche Diagnostics, Mannheim, Germany) by an improved nick translation procedure (for details see [25]). The reaction time was 55 min at 15 °C. To obtain a specific H3 histone probe for *A. anguillae*, fragments of histone H3 genes were amplified using degenerate primers H3aF (5′–ATGGCTCGTACCAAGCAGAC(ACG)GC–3′) and H3aR (5′–ATATCCTT(AG)GGCAT(AG)AT(AG)GTG AC–3′) [10] and gDNA of *A. anguillae* as template. The product obtained by PCR was cloned by ligation into the Promega pGem T-Easy vector (Promega), purified using the NucleoSpinPlasmid kit (Macherey-Nagel, Düren, Germany), sequenced (SEQme, Dobříš, Czech Republic), and confirmed as a histone gene by BLAST search. The verified H3 histone sequence was deposited in GenBank under the accession number OR000720. A new specific pair of primers was designed for the verified sequence using the Geneious Prime version 2021.1.1 software, AAH3F 5′–ACTGTTGCGCTGAGGGAAAT–3′ and AAH3R 5′–ACGACTCACATGCTTCCTGG–3′. Using this primer set, a 160-bp-long H3 sequence was amplified by PCR under the following conditions: initial denaturation step at 95 °C for 3 min, followed by 30 cycles of denaturation at 94 °C for 15 s, annealing at 59 °C for 30 s, and extension at 72 °C for 60 s, and a final extension at 72 °C for 10 min. The H3 fragments were purified using the Wizard SV Gel and PCR Clean-Up System and used as template DNA to prepare labeled probes by PCR with biotin-16-dUTP. The labeled probes were checked on a 1% agarose gel in TAE buffer.

### Fluorescence *in situ* hybridization (FISH)

The one- and two-color FISH experiments were performed according to the protocol previously used for cestode species [45], with some modifications according to the improved protocol [9]. Chromosome slides were removed from the freezer, dehydrated in graded ethanol series, and air-dried. The slides were pretreated with 100 μg/mL RNase A for 1 h at 37 °C in a humid chamber to remove RNA, washed twice in 2 × SSC (saline-sodium citrate) for 5 min each at RT, and then blocked in 5 × Denhardt’s solution for 30 min at 37 °C. In the next step, chromosomal DNA on the slides was denatured in 70% formamide for 3.5 min at 68 °C. The hybridization mixture for each slide (10 μL: 50% deionized formamide, 10% dextran sulfate, 2 × SSC) contained ∼ 35 ng of biotinylated or DIG-labeled 18S rDNA probe or ∼ 50 ng of biotinylated H3 histone probe and 25 μg of sonicated salmon sperm DNA (Sigma-Aldrich, St. Louis, MO, USA). The probe was denatured at 90 °C for 5 min in a water bath, dropped onto the slides, which were sealed and hybridized overnight at 37 °C in a humid chamber. FISH experiments were performed as a one-color FISH in the HN (18S rDNA, H3) and OL (18S rDNA) populations and as a two-color FISH (18S rDNA/H3) in the ZŠ population. The first wash after hybridization was performed five times with 2 × SSC for 2 min at 46 °C, the next wash was performed twice for 5 min with 0.1 × SSC at 62 °C, and the last wash was performed at RT in 4 × SSC with 0.1% Tween 20. Biotinylated probes were detected with Cy3-conjugated streptavidin (Jackson ImmunoRes Labs. Inc., West Grove, PA, USA) and amplified with biotinylated anti-streptavidin (Vector Labs. Inc., Burlingame, CA, USA), which was in turn detected with Cy3-conjugated streptavidin. DIG-labeled probes were detected with anti-digoxigenin-FITC (Sigma-Aldrich). Chromosomes were counterstained with DAPI in ProLong Antifade Medium (Invitrogen, Carlsbad, CA, USA).

### Microscopy and image processing

Stained slides were analyzed using a combined light and fluorescence microscope LEICA DM 4000 B equipped with a DFC 450 C digital camera. Fluorescence images were captured in grayscale, pseudo-colored, merged, and brightness and contrast were optimized using Adobe Photoshop, version 7.0.

## Results

### Karyotype and course of meiosis

**Figure 1:**
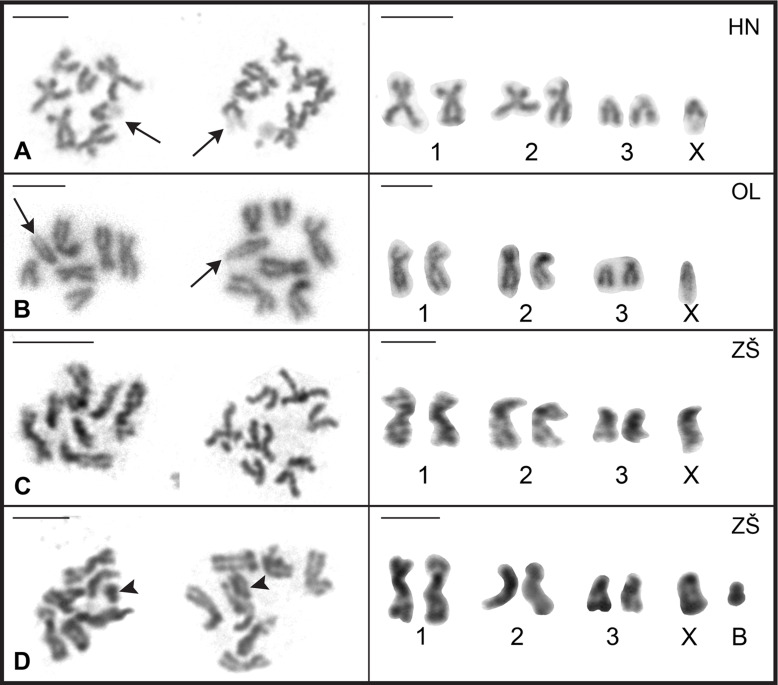
Two mitotic metaphases (left panel) and karyotypes (right panel) of *Acanthocephalus anguillae* males*.* **A** HN population, **B** OL population, **C** ZŠ population, **D** ZŠ population showing a B chromosome (arrowhead). Note a weakly stained region on X chromosomes in HN and OL population (arrow). Bar = 5 μm.

**Figure 2:**
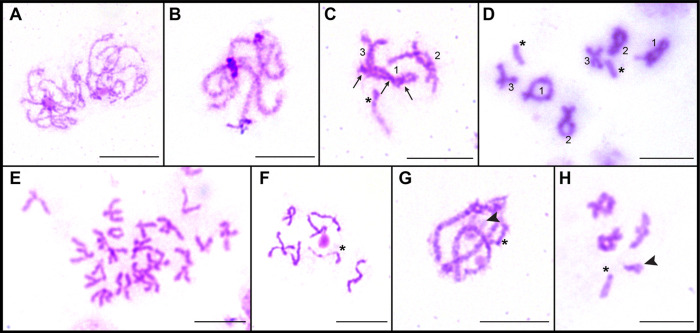
Meiotic divisions of spermatocytes of *Acanthocephalus anguillae* stained with Giemsa. **A** Zygotene. **B** Pachytene. **C** Early diplotene with three chiasmata in the longest bivalent (arrows), representing the largest chromosome pair No. 1. **D** Late diplotene, two cells. **E** Metaphase I bivalents, more cells. **F** Late metaphase II. **G** Pachytene nucleus with one B chromosome (ZŠ population). **H** Diplotene nucleus with one B chromosome (ZŠ population). Asterisks indicate the X chromosome; arrowheads in **G, H** indicate the B chromosome. Bar = 10 μm.

Karyotype analysis of *A. anguillae* was performed on 249 well-spread oogonial and spermatogonial mitotic metaphase plates (63 metaphases from ZŠ, 47♂/16♀; 108 from OL, 83♂/25♀; 78 from HN, 61♂/17♀). The chromosome complement of all populations consisted of three autosome pairs and two X chromosomes in females or one X chromosome in males. The representative mitotic metaphase images of the males and the composite karyotypes of the three studied populations of *A. anguillae* are shown in Figure 1. Autosomes in all three populations gradually decrease in size, and their morphology is identical; pair 1 is metacentric (m), pair 2 is meta-submetacentric (m-sm), and pair 3 is acrocentric (a). The term meta-submetacentric was used when the centromeric index reached the threshold value for determining the type of chromosome morphology. The karyotype formula was determined as 2*n* = 7/8; *n* = 1m + 1m-sm + 1a + 1a (X). Comparison of their absolute length showed very similar values in populations HN and OL, autosomes of population ZŠ were slightly longer. The X chromosome is acrocentric in each population and the sex chromosome system is X0 in males and XX in females. In addition, all metaphase spreads of HN and OL specimens examined had a distinct, long and lightly stained region of uncondensed DNA on the long arms of the X chromosomes (Figs. 1A and 1B), whereas this region was much less evident or not observed at all in the metaphase spreads of ZŠ (Figs. 1C and 1D). The chromosomes of the ZŠ population were generally more difficult to evaluate compared to the chromosomes of the HN and OL populations, which is also clearly seen in the representative mitotic metaphase images in Figure 1. Of the 35 specimens examined from the ZŠ population, 12 (34%) carried a small, supernumerary B chromosome. The presence of the B chromosome was independent of sex and was always one per cell (Fig. 1D), but was not present in all cells of each specimen (intraindividual variation) (Fig. 1C). The B chromosome was smaller than the smallest chromosomes of the standard complement. Meiotic spermatocytes examined in the testes showed the standard steps of cell division characteristic of eukaryotic organisms in each population, and most specimens yielded readily analyzable meiotic plates. The pachytene and diplotene nuclei (Fig. 2) were sufficiently spread to allow counting and accurate identification of individual bivalents and the X chromosome univalent (Fig. 2, asterisks). At the diplotene stage, early diplotene (Fig. 2C), and late diplotene/diakinesis (Figs. 2D and 2H), the distribution of chiasmata in the three autosomal bivalents was evident; the largest bivalent usually had two or three chiasmata in all cells evaluated. The B chromosome was detected not only in mitotic, but also in meiotic cells. Some pachytene, but especially diplotene/diakinesis nuclei were amenable to the analysis of the B chromosome presence (Figs. 2G and 2H). During meiosis, the B chromosome was always present as a univalent indicating that there is no association or pairing with the A complement.

A summary of the absolute and relative lengths, centromeric indices, and classification of chromosome pairs obtained after measuring ten Giemsa-stained metaphase plates for each of the three populations is given in Table 1. There were slight differences in the absolute length of individual chromosome pairs among all three populations; overall, chromosomes were longest in the population of ZŠ. Gross chromosome morphology based on centromeric indices was consistent. Comparative examination of the relative lengths of the corresponding chromosomes revealed no significant differences between the populations (*p* > 0.05).

**Table 1:** Measurement (mean ± SD) and classification of chromosomes in males of *Acanthocephalus anguillae.*

Chromosome number		Absolute length (mean ± SD^A^) (μm)	Relative length (mean ± SD) (%)	Centromeric index (mean ± SD)	Classification^b^
1	Hnilec (HN)	5.33 ± 0.22	32.64 ± 1.32	45.55 ± 1.09	m
	Olšava (OL)	5.23 ± 0.11	31.03 ± 0.92	44.84 ± 0.92	m
	ZŠ	5.78 ± 0.16	32.63 ± 0.93	47.12 ± 0.94	m
2	Hnilec	4.72 ± 0.21	28.87 ± 1.29	40.27 ± 1.19	m-sm
	Olšava	4.75 ± 0.17	28.20 ± 1.01	39.20 ± 1.32	m-sm
	ZŠ	5.07 ± 0.14	28.61 ± 0.81	39.97 ± 0.96	m-sm
3	Hnilec	3.00 ± 0.14	18.37 ± 0.83	19.24 ± 2.04	a
	Olšava	3.05 ± 0.18	18.10 ± 1.09	24.60 ± 0.58	a
	ZŠ	3.44 ± 0.07	19.40 ± 0.39	24.35 ± 1.17	a
X	Hnilec	3.29 ± 0.14	20.13 ± 1.12	9.41 ± 1.79	a
	Olšava	3.82 ± 0.16	22.68 ± 0.94	9.80 ± 1.21	a
	ZŠ	3.43 ± 0.20	19.37 ± 1.12	14.67 ± 1.71	a

Study site: HN – Hnilec River, OL – Olšava River, ZŠ – Zemplínska Šírava reservoir.

a Mean absolute lengths were calculated from 10 best mitotic metaphase for each *A. anguillae* population.

b Classification according to Dos Santos Guerra [17], a – acrocentric; m – metacentric, sm – submetacentric chromosome pair, m-sm – was used when the centromeric index reached the threshold value for the type of morphological determination of the chromosome.

### Mapping of 18S rDNA and H3 genes

**Figure 3:**
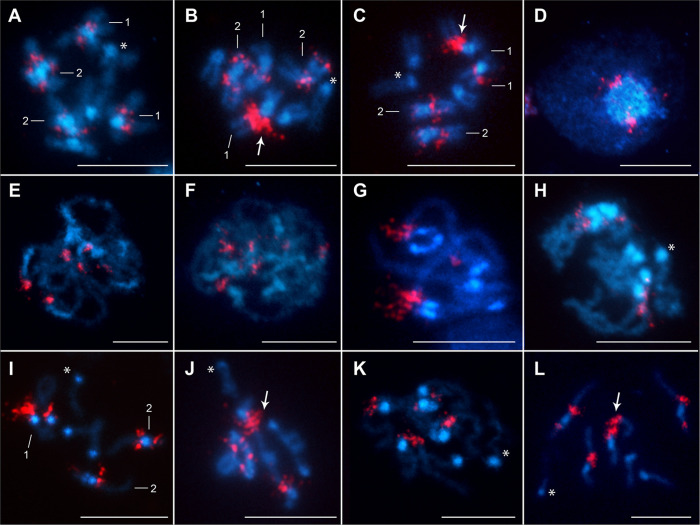
FISH with the 18S rDNA probe (red) on chromosomes of *Acanthocephalus anguillae* males from HN and OL rivers. **A** Mitotic metaphase (HN). **B, C** Mitotic metaphases (OL). **D** Interphase nucleus. **E** Pachytene (HN). **F** Zygotene and **G** pachytene bivalents showing clusters of interstitial 18S rDNA signals associated with DAPI-positive heterochromatic blocks (OL). **H** Diplotene (HN). **I** Diplotene/diakinesis with already separated bivalents No. 2 and **J** early anaphase with the largest bivalents almost separated (OL). **K** Metaphase I (HN). **L** Metaphase II (OL). Note the larger signals of the 18S rDNA probe on a homologous chromosome of pair No. 1 in the OL population, indicated by arrows. Asterisks indicate the X chromosome. Chromosomes were counterstained with DAPI. Bar = 10 μm.

**Figure 4:**
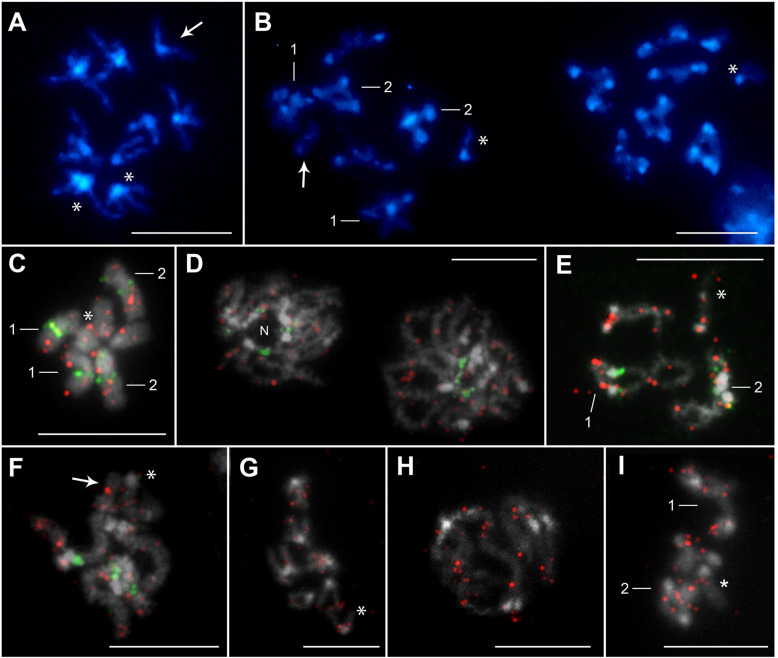
Distribution of heterochromatin after DAPI-staining (blue; ZŠ population) (**A–B**), two-color FISH with 18S rDNA (green) and H3 histone (red) probes on chromosomes of *Acanthocephalus anguillae* males from the ZŠ population (**C–F**) and FISH with H3 histone probe (red) on chromosomes of *A. anguillae* males from the HN population (**G–I**). **A** Mitotic metaphase with B chromosome and **B** two anaphase nuclei, left with and right without B chromosome. **C** Mitotic metaphase. **D** Two pachytene nuclei, the left with nucleolus residue (N). **E** Diplotene. **F** Pachytene with B chromosome. **G** Mitotic metaphase. **H** Pachytene. **I** Diplotene. Arrows indicate the B chromosome and asterisks indicate the X chromosome. Bar = 10 μm.

Chromosomal mapping revealed the presence of 18S rDNA clusters at three loci in each population examined (Figs. 3 and 4). In all cases, the FISH signals were located on the first two autosome pairs, one on pair No. 1 and two on pair No. 2. No signal for 18S rDNA was observed on the B chromosome. On the metacentric chromosome pair No. 1, the signals were always located in the interstitial region on the long arm near the centromere. The size and intensity of signals differed markedly between the homologous chromosomes of pair No. 1 in the OL population. The FISH signal was always stronger on one of the homologous chromosomes than on the other, which was clearly visible during meiotic division (Figs. 3B, 3C, 3I, 3J, 3L). This size heteromorphism was not observed in the HN (Fig. 3A) and ZŠ (Fig. 4) populations. On the second chromosome pair, two interstitial clusters of rDNA genes were detected near the centromere on short and long arms. The same location of rDNA clusters was observed in all three populations. The hybridization signals were colocalized with a strong block of DAPI-highlighted heterochromatin in the HN and OL populations (Fig. 3). The DAPI-positive A + T-rich heterochromatin blocks were concentrated in the centromeric region of all chromosomes. In addition, the pericentromeric heterochromatin blocks in chromosome No. 2 were extended to the long arms in the HN and OL populations (Fig. 3). In the ZŠ population, the blocks of DAPI-positive heterochromatin were also detected in the centromeric region on all chromosomes (Fig. 4). A much less bright but visible band was also detected on the B chromosome (Figs. 4A and 4B). No other heterochromatin bands were visible on the chromosomes of all three populations. FISH with the H3 histone probe revealed multiple hybridization signals, indicating that clusters of the H3 histone genes are dispersed on all chromosomes (Figs. 4C–4I). These signals were well observed in mitotic metaphase nuclei (Figs. 4C and 4G) and meiotic cells with more loose chromosomes during prophase I (Figs. 4, 4D–4F, 4H, 4I). In all individuals, H3 gene signals were detected on all autosomes and the X chromosome. In the ZŠ population, there were also signals on the B chromosomes, but they were much weaker and not always present or possibly not constantly detectable, probably because of the smaller size of the H3 histone gene clusters.

**Figure 5:**
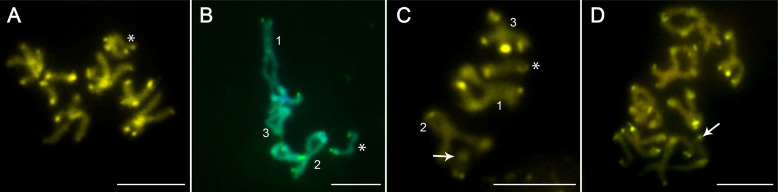
Chromosomes of *Acanthocephalus anguillae* males stained with chromomycin A_3_/Methyl Green. **A** Late mitotic metaphase (HN population). **B** Merged chromomycin A_3_/DAPI image of a diplotene nucleus (OL population). **C, D** Diplotene nuclei with B chromosome (ZŠ population). Arrows indicate the B chromosome and asterisks the X chromosome. Bar = 10 μm.

CMA_3_/Methyl Green staining, revealing the presence of GC-rich heterochromatin blocks, showed positive signals in the terminal – telomeric regions on all chromosomes in each of the three populations examined (Fig. 5). The B chromosome also carried small GC-rich blocks at the chromosome ends (Figs. 5C and 5D).

## Discussion

The low chromosome number in Acanthocephala, the presence of sex chromosomes and, as shown in our work, easy and clear mitotic and meiotic chromosome spreads with the possibility to study the exact chromosome morphology, make this group of intestinal parasites very interesting for cytogenetic studies of karyotype evolution. In addition, the obtained karyological data can also be helpful in solving taxonomic problems, which is of great importance for this group of parasites.

To date, our knowledge of acanthocephalan chromosomes is limited to 13 studied species, including the first karyotype description of *A. anguillae* in this paper. Detailed descriptions of the chromosome complement are known for eight species, and only information on chromosome number is available for the other five (see Supplementary Table S1 for details). *Acanthocephalus anguillae* belongs to the order Echinorhynchida, which is the best studied of the Acanthocephala with nine species examined cytogenetically. Based on the very limited data available, at least one karyological feature of the order Echinorhynchida is evident, namely the small and stable number of chromosomes (2*n* = 7/8). The karyotypes of *Echinorhynchus gadi* Zoega in Müller, 1776 and *A. ranae* were described with twice as many chromosomes, *i.e.*, with 2*n* = 16, but no information on sex and karyotype structure was provided. However, it should be emphasized here that these data are from Hamann’s first records of 1891 [24] and were merely adopted by Walton [77]. It is likely that chromosome numbers evaluated in early studies of chromosomes on squash preparations were incorrectly estimated, *e.g.*, by counting together the chromosomes of two adjacent cells, although the whole-genome duplication cannot be completely ruled out [59, 80]. The later study of *A. ranae* [29] reported a diploid number of 2*n* = 8 in females. A different diploid number, *n* = 5/6, was reported only for *Leptorhynchoides thecatus* (Linton, 1891) [5], while the closely related *L. plagicephalus* (Westrumb, 1821) has 2*n* = 7/8 [19].

The karyotypes of *A. anguillae* from three distant Slovak localities showed the same number of 2*n* = 7/8, confirming the assumption of a stable low chromosome number in the order Echinorhynchida, in contrast to higher modal chromosome numbers in other groups of parasitic worms, such as 2*n* = 14 in the Monogenea [35] and 2*n* = 28 in the Cestoda and the Trematoda [48, 70]. An identically low diploid number was observed in the congeneric species *A. lucii* in two geographically distant populations, one from Bulgaria and one from Slovakia [42, 68]. In all studied populations of *A. lucii* and *A. anguillae*, the particular karyotype characteristics (chromosome and relative length) of the corresponding chromosome pairs and TCL differ slightly, which we associate with the different degree of chromosome spiralization on the evaluated metaphase plates. Chromosome length and TCL are important karyotype characteristics but should be compared with caution. Chromosomes of *A. lucii* were classified as submetacentric and subtelocentric according to the system of Levan *et al.* [36], which based on CI values [42, 68] corresponds to submetacentric and acrocentric chromosomes according to the nomenclature [17] used in our study. Obvious interspecific and also interpopulation karyotype variations were observed in the morphology of the third chromosome pair, which is submetacentric in *A. lucii* from Slovakia [68], while it is acrocentric (subtelocentric) in *A. lucii* from Bulgaria [42] and *A. anguillae* in our study. Thus, the chromosome sets of *A. anguillae* (this study) and the *A. lucii* population from Bulgaria appear to be closer. Given the great similarities between the karyotypes of these two species with the same chromosome number, the simplest and most reasonable assumption is that the shape of chromosome No. 3 has been altered by small intrachromosomal rearrangements, the pericentric inversion. This type of chromosome rearrangement has also been proposed as a differentiation mechanism in other parasite groups such as trematodes and cestodes [27, 46]. In the third thoroughly studied species of this genus, *A. ranae*, the same diploid number 2*n* = 8, but only one type of chromosome shape, all metacentric, was documented in the female specimens [29]. Chromosome lengths are not listed. In this early work, the male sex chromosome system was determined to be heterogametic (XY) and the female homogametic (XX). However, based on the published images and their evaluation from today’s perspective and knowledge, the sex system was incorrectly determined and the chromosomal morphology is highly questionable and needs to be reevaluated using current methods.

Evolutionary changes in the karyotype, such as chromosomal rearrangements and polyploidy, support genetic differentiation of organisms [33]. Pericentric inversion involved in the evolution of chromosome No. 2 has contributed to the diversification of the closely related and morphologically very similar acanthocephalan species *Pomphorhynchus tereticollis* and *P. laevis* [3]. Two different rDNA clusters were found in these species, located on the two largest chromosome pairs. Their karyotypes differ in the position of the nucleolus organizer region (NOR) and also in the position of the centromere on chromosome pair number 2. Two successive pericentric inversions, one shifting the position of the NOR and the next shifting the centromere, have been proposed as a hypothetical scenario for chromosome rearrangement (see Figure 5 in [3]). These two *Pomphorhynchus* species, which belong to the family Pomphorhynchidae, are all Acanthocephala species that have been studied so far using FISH. In the family Echinorhynchidae, the present study demonstrated that specimens of *A. anguillae* have three rRNA gene clusters per haploid genome, located interstitially, near the centromere on the first two chromosome pairs. Thus, *A. anguillae* has the same chromosome number (2*n* = 7/8) and also the interstitial placement of the 18S loci on the first two autosomes, but one locus more in contrast to the *Pomphorhynchus* species. The rDNA sequences are conserved, but their chromosomal distribution is a source of species differentiation [57, 63]. It is known that rDNA can spread between or within the chromosome and that the increase or decrease of the number of rDNA sites can be caused by retroelement-mediated rDNA transpositions [64] and by chromosomal rearrangements such as inversion or translocation. The fragmentary information does not allow us to speculate whether and what changes in the number and chromosomal positions of the NORs have occurred. Very cautiously, it could be said that Echinorhynchida species have a preference for interstitial, autosomal rRNA gene positioning. However, the different patterns on the chromosome in only three acanthocephalan species studied could indicate frequent changes in chromosomal rDNA distribution and a possible importance of rDNAs in genome specification. We also obtained the first FISH data on the distribution of H3 histone genes in Acanthocephala. In contrast to the 18S rDNA, FISH with the H3 histone probe revealed a scattered pattern of this sequence in the genomes of two *A. anguillae* populations, as we detected scattered signals in all chromosomes (with even more clusters on individual chromosomes), including the sex chromosomes and, interestingly, in the ZŠ population also the B chromosomes. Although histone genes are typically organized in clusters, exceptions exist. For example, in fish species *Characidium zebra* and *C. gomesi*, the histones H1 and H4 are dispersed throughout the genome and are associated with mobile elements, although the role of mobile element as the dispersing force has not been proven [55]. Similarly, in the spot-legged wood turtle (*Rhinoclemmys punctularia*), the H3 genes are organized both in large clusters and scattered copies and contain an integrated part of the Gypsy element [13]. In seven Paradontidae species, it has been shown that H1 genes are scattered, while H4 genes form large clusters. Interestingly, the H1 genes were associated with a mobile element, but the H4 genes were not [74]. These data suggest that the mobile elements may be the evolutionary force which distributed the genes throughout the genome. In this work, we isolated a fragment of histone H3 gene with no adjacent sequence of a mobile element, but its presence is possible in flanking regions of this fragment. Therefore, reconstruction of entire region is needed to elucidate the mechanism responsible for histone gene spreading.

### B chromosomes

The most striking difference between the three studied Slovak populations of *A. anguillae* is the presence of a supernumerary B chromosome in the ZŠ population, while it is absent in the other two populations. B chromosomes were also observed in the Slovak *A. lucii* population, which is the main difference between the Slovak and Bulgarian worms, along with the morphology of chromosome No. 3. In both species, the B chromosomes were always the smallest in complement, but differed in their morphology, which was acrocentric in *A. anguillae*, but metacentric in *A. lucii*. Their number varied from one to five in the karyotype of *A. lucii*, with 85% of the worms examined having them [68]. In the karyotype of *A. anguillae*, the occurrence was less frequent and only in the ZŠ population, reaching 34%. In both species, the B chromosomes did not pair with any of the A chromosomes.

The B chromosomes are known to be non-essential supernumerary elements present in thousands of plant, animal, and fungal species [32] and have also been described in some digenean parasites [21, 69]. Comparison of their molecular composition has shown that most DNA sequences found on B chromosomes are also present on A chromosomes [12], and although mainly repetitive DNA is found on them [8], protein-coding genes have also been discovered [39]. Widely accepted pathways by which B chromosomes arise include intraspecific derivation from the A chromosomes (autosomes or sex chromosomes) or interspecific hybridization [11]. In addition, however, by-products of structural chromosome rearrangements could also be candidates for their origin. The scenario that B chromosomes arose from chromosome rearrangements followed by amplification of DNA sequences has been proposed for some plant species as the result of allopolyploidization [15, 31]. A similar scenario, the origin of B chromosomes due to chromosome breaks but due to environmental pollution, has been proposed in studies of cichlids [18, 51]. Pollution usually has obvious and severe negative effects on chromosomes. A pilot study on the effects of water pollution in Zemplínska Šírava on the chromosomes of the parasite *Caryophyllaeus laticeps* clearly shows this effect [46]. An increased incidence of chromosomal aberrations was observed, although no B chromosomes were detected in this species. However, the chromosomes of *A. anguillae* are quite small and their morphology in specimens from Zemplínska Šírava was strange, possibly affected by contamination, so the evaluation of chromosomal aberrations was not possible. The present work provides the second evidence for the presence of B chromosomes in the species of the genus *Acanthocephalus* originating from heavily polluted sites (cf. [68]), whereas no B chromosomes were detected in the karyological analysis of these species from unpolluted sites. Mutagenic heavy metals and PCBs in the aquatic environment could be the culprits. Therefore, it is likely that induced breaks in the A chromosomes could generate chromosome fragments or DNA sequences that have the potential to form B chromosomes. The well-known bioaccumulation ability of multicellular intestinal parasites of fish, especially acanthocephalan worms, which are able to accumulate significantly higher amounts of heavy metals and PCBs compared to their hosts [7, 40, 66, 75], could facilitate the karyological changes in *A. anguillae*. A possible link between unfavorable environmental conditions and the presence of mutagenic pollutants and the occurrence of B chromosomes was also noted by Špakulová *et al.* [68] in the population of *A. lucii* from the heavily polluted Ružín reservoir in eastern Slovakia and for some digenean flukes [2].

B chromosomes are typically completely or partially heterochromatic [15]. Our results show that the B chromosome in *A. anguillae* has a low enrichment of CMA_3_/DAPI heterochromatin, with A + T-rich heterochromatin concentrated in C-positive regions and a very small but detectable amount of G + C-rich heterochromatin restricted to telomeric regions. Chromosomal mapping of multigene families (18S rDNA, 5S rDNA, histone H3 genes or U1/U2 snDNA) provided remarkable information about the possible autosomal origin/evolution of B chromosomes due to their common location in B and A chromosomes [8, 43, 72]. Our results showed the presence of histone H3 genes on the B chromosome, but also the distribution of H3 histone sequences on all autosomes and the X chromosome, so these sequences, as well as the mapping of the 18 s rDNA probe, are not informative in the case of *A. anguillae*. The fact that this B chromosome does not show strong heterochromatization may indicate its recent origin.

Our study is only the second work to provide information on the number and location of rRNA genes, and the first on histone H3 genes throughout the phylum Acanthocephala. Chromosomal changes/rearrangements may be crucial for the speciation process. As the case of the *Pomphorhynchus* species shows, speciation was triggered by geographic factors and reinforced by chromosomal rearrangements – pericentric inversion. FISH with the 18S rDNA probe looks particularly promising, although it is not sufficient on its own. H3 histone genes are scattered in the species we studied, but this does not necessarily mean that this is the case in other acanthocephalan species. Therefore, comparative chromosome research, including basic karyotype analyses, mapping of genes and repetitive sequences is needed in a variety of acanthocephalan species. In addition, there are still many unresolved problems in phylogeny and taxonomy at the family and genus levels, and karyological analysis can help clarify some of these problems. This study also provided new information on the presence of B chromosomes in the genus *Acanthocephalus*, possibly related to the long-term PCB contamination of the Zemplínska Šírava waters.

## Supplementary material

The supplementary material of this article is available at https://www.parasite-journal.org/10.1051/parasite/2023045/olm. *Supplementary Table S1*: Summary of karyotype data of acanthocephalan species.

## References

[1] Amin OM. 2013. Classification of the Acanthocephala. Folia Parasitologica, 60, 273–305. 24261131 10.14411/fp.2013.031

[2] Baršiené J. 1993. The chromosome sets of trematodes. Parazitologiya, 27, 336–353. 8414652

[3] Bombarová M, Marec F, Nguyen P, Špakulová M. 2007. Divergent location of ribosomal genes in chromosomes of fish thorny-headed worms, *Pomphorhynchus laevis* and *Pomphorhynchus tereticollis* (Acanthocephala). Genetica, 131, 141–149. 17143651 10.1007/s10709-006-9124-3

[4] Bombarová M, Vítková M, Špakulová M, Koubková B. 2009. Telomere analysis of platyhelminths and acanthocephalans by FISH and Southern hybridization. Genome, 52, 897–903. 19935913 10.1139/g09-063

[5] Bone LW. 1974. The chromosomes of *Leptorhynchoides thecatus* (Acanthocephala). Journal of Parasitology, 60, 818. 4430949

[6] Bone LW. 1974. The chromosomes of *Neoechinorhynchus cylindratus* (Acanthocephala). Journal of Parasitology, 60, 731–732.

[7] Brázová T, Miklisová D, Barčák D, Uhrovič D, Šalamún P, Orosová M, Oros M. 2021. Hazardous pollutants in the environment: Fish host-parasite interactions and bioaccumulation of polychlorinated biphenyls. Environmental Pollution, 291, 118175. 34543958 10.1016/j.envpol.2021.118175

[8] Bueno D, Palacios-Gimenez OM, Cabral-de-Mello DC. 2013. Chromosomal mapping of repetitive DNAs in the grasshopper *Abracris flavolineata* reveal possible ancestry of the B chromosome and H3 histone spreading. PLoS One, 8, e66532. 23826099 10.1371/journal.pone.0066532 PMC3694960

[9] Cabral-de-Mello DC, Marec F. 2021. Universal fluorescence in situ hybridization (FISH) protocol for mapping repetitive DNAs in insects and other arthropods. Molecular Genetics and Genomics, 296, 513–526. 33625598 10.1007/s00438-021-01765-2

[10] Cabrero J, López-León M, Teruel M, Camacho JPM. 2009. Chromosome mapping of H3 and H4 histone gene clusters in 35 species of acridid grasshoppers. Chromosome Research, 17, 397–404. 19337846 10.1007/s10577-009-9030-5

[11] Camacho JP, Sharbel TF, Beukeboom LW. 2000. B-chromosome evolution. Philosophical Transactions of the Royal Society B: Biological Sciences, 355, 163–178. 10.1098/rstb.2000.0556 PMC1692730 10724453

[12] Camacho JPM. 2005. B Chromosomes, in The Evolution of the Genome. Gregory TR, Editor. Elsevier Academic Press. p. 223–286.

[13] Cavalcante MG, Souza LF, Vicari MR, Matos de Bastos CE, Viana de Sousa J, Nagamachi CY, Pieczarka JC, Martins C, Rodrigues Noronha RC. 2020. Molecular cytogenetics characterization of *Rhinoclemmys punctularia* (Testudines, Geoemydidae) and description of a Gypsy-H3 association in its genome. Gene, 738, 144477. 32061764 10.1016/j.gene.2020.144477

[14] Chmúrčiaková N, Kašný M, Orosová M. 2020. Cytogenetics of *Eudiplozoon nipponicum* (Monogenea, Diplozoidae): Karyotype, spermatocyte division and 18S rDNA location. Parasitology International, 76, 102031. 31770588 10.1016/j.parint.2019.102031

[15] Dhar MK, Friebe B, Koul AK, Bikram SG. 2002. Origin of an apparent B chromosome by mutation, chromosome fragmentation and specific DNA sequence amplification. Chromosoma, 111, 332–340. 12474062 10.1007/s00412-002-0214-4

[16] Dobigny G, Terence JF, Robinson J, Volobouev V. 2004. Cytogenetics and cladistics. Systematic Biology, 53, 470–484. 15503674 10.1080/10635150490445698

[17] Guerra Dos Santos. 1986. Reviewing the chromosomes nomenclature of Levan et al. Revista Brasileira de Genetica, 9, 741–743.

[18] Feldberg E, Porto JIR, Alves-Brinn MN, Mendonça MNC, Benzaquem DC. 2004. B chromosomes in Amazonian cichlid species. Cytogenetic and Genome Research, 106, 195–198. 15292591 10.1159/000079287

[19] Fontana F, Dezfuli BS, Benvenuti M. 1993a. The chromosome complement of *Leptorhyncoides plagicephalus* (Westrumb, 1821), (Acanthocephala: Rhadinorhynchidae). Cytologia, 58, 393–396.

[20] Fontana F, Dezfuli BS, Benvenuti M. 1993b. Somatic and meiotic chromosomes in male and female of *Pomphorhynchus laevis* Müller, 1776 (Acanthocephala: Pomphorhynchidae). Caryologia, 46, 329–334.

[21] García-Souto D, Pasantes JJ. 2015. Molecular cytogenetics in digenean parasites: Linked and unlinked major and 5S rDNAs, B Chromosomes and karyotype diversification. Cytogenetic and Genome Research, 147, 195–207. 26680763 10.1159/000442504

[22] García-Varela M, Pérez-Ponce de León G, Torre P, Cummings MP, Sarma SSS, Laclette JP. 2000. Phylogenetic relationships of Acanthocephala based on analysis of 18S ribosomal RNA gene sequences. Journal of Molecular Evolution, 50, 532–540. 10835483 10.1007/s002390010056

[23] García-Varela M, Andrade-Gómez L. 2021. First steps to understand the systematics of Echinorhynchidae Cobbold, 1876 (Acanthocephala), inferred through nuclear gene sequences. Parasitology International, 81, 102264. 33301950 10.1016/j.parint.2020.102264

[24] Hamann O. 1891. Monographie der Acanthocephalen (Echinorhynchen). Ihre Entwicklungsgeschichte, Histogenie und Anatomie, nebst Beitragen zur Systematik und Biologie. Jenaische Zeitschrift für Naturwissenschaft, 25, 113–231.

[25] Hejníčková M, Dalíková M, Potocký P, Tammaru T, Trehubenko M, Kubíčková M, Marec F, Zrzavá M. 2021. Degenerated, undifferentiated, rearranged, lost: High variability of sex chromosomes in Geometridae (Lepidoptera) identified by sex chromatin. Cells, 10, 2230. 34571879 10.3390/cells10092230 PMC8468057

[26] Hirai H. 2014. Chromosomal differentiation of schistosomes: What is the message? Frontiers in Genetics, 5, 00301. 10.3389/fgene.2014.00301 PMC4157585 25250045

[27] Hirai H, Taguchi T, Saitoh M, Kawanaka M, Sugiyama H, Habe S. 2000. Chromosomal differentiation of the *Schistosoma japonicum* complex. International Journal for Parasitology, 30, 441–452. 10731567 10.1016/s0020-7519(99)00186-1

[28] Huston DC, Cribb TH, Smales LR. 2020. Molecular characterization of acanthocephalans from Australian marine teleosts: proposal of a new family, synonymy of another and transfer of taxa between orders. Systematic Parasitology, 97, 1–23. 31912420 10.1007/s11230-019-09896-2

[29] John B. 1957. The chromosomes of zooparasites I. *Acanthocephalus ranae* (Acanthocephala: Echinorhynchidae). Chromosoma, 8, 730–738. 13523750

[30] Jones AW, Ward HL. 1950. The chromosomes of *Macracanthorhynchus hirudinaceous* (Pallas). Journal of Parasitology, 36, 86. 15409580

[31] Jones N, Houben A. 2003. B chromosomes in plants: escapees from the A chromosome genome? Trends in Plant Science, 8, 417–423. 13678908 10.1016/S1360-1385(03)00187-0

[32] Jones RN. 2018. Transmission and drive involving parasitic B chromosomes. Genes, 9, 388. 30065230 10.3390/genes9080388 PMC6115934

[33] Kellogg EA. 2016. Has the connection between polyploidy and diversification actually been tested? Current Opinion in Plant Biology, 30, 25–32. 26855304 10.1016/j.pbi.2016.01.002

[34] Kennedy CR. 2006. Ecology of the Acanthocephala, 1st edn. Cambridge University Press.

[35] Košková E, Špakulová M, Koubková B, Reblánová M, Orosová M. 2011. Comparative karyological analysis of four diplozoid species (Monogenea, Diplozoidae), gill parasites of cyprinid fishes. Parasitology Research, 108, 935–941. 20981442 10.1007/s00436-010-2135-0

[36] Levan A, Fredga K, Sandberg A. 1964. Nomenclature for centromere position on chromosomes. Hereditas, 52, 201–220.

[37] Liehr T. 2021. Molecular cytogenetics in the era of chromosomics and cytogenomic approaches. Frontiers in Genetics, 12, 720507. 34721522 10.3389/fgene.2021.720507 PMC8548727

[38] Lisitsyna OI. 2019. Fauna of Ukraine, vol 31. Acanthocephala. Kiev. p. 223.

[39] Milani D, Ruiz-Ruano FJ, Camacho JPM, Cabral-de-Mello DC. 2021. Out of patterns, the euchromatic B chromosome of the grasshopper *Abracris flavolineata* is not enriched in high-copy repeats. Heredity, 127, 475–483. 34482369 10.1038/s41437-021-00470-5 PMC8551250

[40] Molbert N, Alliot F, Leroux-Coyau M, Médoc V, Biard C, Meylan S, Jacquin L, Santos R, Goutte A. 2020. Potential benefits of acanthocephalan parasites for chub hosts in polluted environments. Environmental Science & Technology, 54, 5540–5549. 32267695 10.1021/acs.est.0c00177

[41] Mutafova T, Nedeva I. 1988. Oogenesis and spermatogenesis of *Pomphorhynchus laevis* (Müller, 1776) (Acanthocephala: Pomphorhynchidae). Khelminthologyia, 25, 23–28.

[42] Mutafova T, Nedeva I, Kanev I. 1997. Chromosomes of *Acanthocephalus lucii*. Journal of Helminthology, 71, 261–262.

[43] Oliveira NL, Cabral-de-Mello DC, Rocha MF, Loreto V, Martins C. 2011. Chromosomal mapping of rDNAs and H3 histone sequences in the grasshopper *Rhammatocerus brasiliensis* (Acrididae, Gomphocerinae): extensive chromosomal dispersion and co-localization of 5S rDNA/H3 histone clusters in the A complement and B chromosome. Molecular Cytogenetics, 4, 24. 22075079 10.1186/1755-8166-4-24 PMC3234176

[44] Orosová M, Špakulová M. 2018. Tapeworm chromosomes: their value in systematics with instructions for cytogenetic study. Folia Parasitologica, 65, 001. 10.14411/fp.2018.001 29528298

[45] Orosová M, Provazníková I, Xi BW, Oros M. 2019. Chromosomal study of *Khawia abbottinae* (Cestoda: Caryophyllidea): karyotype and localization of telomeric and ribosomal sequences after fluorescence in situ hybridization (FISH). Parasitology Research, 118, 2789–2800. 31485863 10.1007/s00436-019-06450-3

[46] Orosová M, Marková A, Marec F, Barčák D, Brázová T, Oros M. 2022. New cytogenetic data on *Caryophyllaeus laticeps* and *Paracaryophyllaeus gotoi*, parasites of evolutionary interest. Parasitology, 149, 1094–1105. 35535487 10.1017/S0031182022000622 PMC11010498

[47] Parenti U, Antoniotti L, Beccio C. 1965. Sex ratio and sex digamety in *Echinorhynchus truttae*. Experientia, 21, 657–658. 5868254 10.1007/BF02144064

[48] Park GM, Im K, Huh S, Yong TS. 2000. Chromosomes of the liver fluke, *Clonorchis sinensis*. Korean Journal of Parasitology, 38, 201–206. 11002660 10.3347/kjp.2000.38.3.201 PMC2728209

[49] Petkevičiūtė R. 1996. A chromosome study of *Schistocephalus solidus* (Müller, 1776) (Cestoda: Pseudophyllidea). Systematic Parasitology, 33, 183–186.

[50] Petkevičiūtė R, Stunženas Stanevičiūtė G. 2011. Clarification of the systematic position of *Cercariaeum crassum* Wesenberg-Lund, 1934 (Digenea), based on karyological analysis and DNA sequences. Journal of Helminthology, 86, 293–301. 21791154 10.1017/S0022149X11000393

[51] Perazzo GX, Noleto RB, Vicari MR, Gava A, Cestari MM. 2018. B chromosome polymorphism in South American cichlid. Neotropical Biodiversity, 4, 3–9.

[52] Percie du Sert N, Hurst V, Ahluwalia A, Alam S, Avey MT, Baker M, Browne WJ, Clark A, Cuthill IC, Dirnagl U, Emerson M, Garner P, Holgate ST, Howells DW, Karp NA, Lazic SE, Lidster K, MacCallum CJ, Macleod M, Pearl EJ, Petersen OH, Rawle F, Reynolds P, Rooney K, Sena ES, Silberberg SD, Steckler T, Würbel H. 2020. The ARRIVE guidelines 2.0: updated guidelines for reporting animal research. PLoS Biology, 18, e3000410. 32663219 10.1371/journal.pbio.3000410 PMC7360023

[53] Perrot-Minnot MJ. 2004. Larval morphology, genetic divergence, and contrasting levels of host manipulation between forms of *Pomphorhynchus laevis* (Acanthocephala). International Journal for Parasitology, 34, 45–54. 14711589 10.1016/j.ijpara.2003.10.005

[54] Perrot-Minnot M-J, Cozzarolo C-S, Amin O, Barčák D, Bauer A, Filipović Marijić V, García-Varela M, Servando Hernández-Orts J, Le Yen TT, Nachev M, Orosová M, Rigaud T, Šariri S, Wattier R, Reyda F, Sures B. 2023. Hooking the scientific community on thorny-headed worms: interesting and exciting facts, knowledge gaps and perspectives for research directions on Acanthocephala. Parasite, 30, 23. 37350678 10.1051/parasite/2023026 PMC10288976

[55] Pucci MB, Nogaroto V, Moreira-Filho O, Vicari MR. 2018. Dispersion of transposable elements and multigene families: Microstructural variation in *Characidium* (Characiformes: Crenuchidae) genomes. Genetics and Molecular Biology, 41, 585–592. 30043833 10.1590/1678-4685-GMB-2017-0121 PMC6136364

[56] Rábová M, Volker M, Pelikánová Š, Ráb P. 2015. Sequential chromosome bandings in fishes, in FISH cytogenetic techniques, ray-fin fishes and chondrichthyans. Ozouf-Costaz C, Pisano E, Foresti F, Toledo LFA, Editors. CRC Press: USA. p. 66–73.

[57] Raskina O, Barber JC, Nevo E, Belyayev A. 2008. Repetitive DNA and chromosomal rearrangements: speciation-related events in plant genomes. Cytogenetic and Genome Research, 120, 351–357. 18504364 10.1159/000121084

[58] Reblánová M, Špakulová M, Orosová M, Králová-Hromadová I, Bazsalovicsová E. 2011. A comparative study of karyotypes and chromosomal location of rDNA genes in important liver flukes *Fasciola hepatica* and *Fascioloides magna* (Trematoda: Fasciolidae). Parasitology Research, 109, 1021–1028. 21509448 10.1007/s00436-011-2339-y

[59] Redmond AK, Casey D, Gundappa MK, Macqueen DJ, McLysaght A. 2023. Independent rediploidization masks shared whole genome duplication in the sturgeon-paddlefish ancestor. Nature Communications, 14, 2879. 10.1038/s41467-023-38714-z PMC10199039 37208359

[60] Ribas TFA, Pieczarka JC, Griffin DK, Kiazim LG, Nagamachi CY, O’Brien PCM, Ferguson-Smith MA, Yang F, Aleixo A, O’Connor RE. 2021. Analysis of multiple chromosomal rearrangements in the genome of *Willisornis vidua* using BAC-FISH and chromosome painting on a supposed conserved karyotype. BMC Ecology and Evolution, 21, 34. 33653261 10.1186/s12862-021-01768-y PMC7927240

[61] Robinson ES. 1964. Chromosome morphology and behavior in *Macracanthorhynchus hirudinaceus*. Journal of Parasitology, 50, 694–697. 14217942

[62] Robinson ES. 1965. The chromosomes of *Moniliformis dubius* (Acanthocephala). Journal of Parasitology, 51, 430–432. 5841339

[63] Roy V, Monti-Dedieu L, Chaminade N, Siljak-Yakovlev S, Aulard S, Lemeunier F, Montchamp-Moreau C. 2005. Evolution of the chromosomal location of rDNA genes in two *Drosophila* species subgroups: *ananassae* and *melanogaster*. Heredity, 94, 388–395. 15726113 10.1038/sj.hdy.6800612

[64] Schubert I. 1984. Mobile nucleolus organizing regions (NORs) in *Allium* (Liliaceae S-Lat)-inferences from the specifity of silver staining. Plant Systematics and Evolution, 144, 291–305.

[65] Stanevičiũté G, Kiseliené V. 2001. Chromosome studies of *Ichthyocotylurus platycephalus* (Creplin, 1825) Odening 1969 with description of triploid variant and comparative karyology of the genus *Ichthyocotylurus*. Parasite, 8, 137–145. 11474981 10.1051/parasite/2001082137

[66] Sures B, Nachev M, Selbach C, Marcogliese DJ. 2017. Parasite responses to pollution: what we know and where we go in ‘Environmental Parasitology’. Parasites & Vectors, 10, 65. 28166838 10.1186/s13071-017-2001-3 PMC5294906

[67] Šalgovičová D, Zmetáková Z. 2006. Polychlorinated biphenyls in muscle tissue of freshwater fish in East Slovakia. Journal of Food and Nutrition Research, 45, 171–178.

[68] Špakulová M, Kráľová-Hromadová I, Dudiňák V, Reddy PV. 2002. Karyotype of *Acanthocephalus lucii:* the first record of supernumerary chromosomes in thorny-headed worms. Parasitology Research, 8, 778–780. 10.1007/s00436-002-0639-y 12122438

[69] Špakulová M, Cassanova JC. 2004. Current knowledge on B chromosomes in natural populations of helminth parasites: a review. Cytogenetic and Genome Research, 106, 222–229. 15292595 10.1159/000079291

[70] Špakulová M, Orosová M, Mackiewicz JS. 2011. Cytogenetics and chromosomes of the tapeworms (Platyhelminthes, Cestoda). Advances in Parasitology, 74, 177–230. 21295678 10.1016/B978-0-12-385897-9.00003-3

[71] Štundlová J, Šmíd J, Nguyen P, Šťáhlavský P. 2019. Cryptic diversity and dynamic chromosome evolution in Alpine scorpions (Euscorpiidae: *Euscorpius*). Molecular and Phylogenetic Evolution, 134, 152–163. 10.1016/j.ympev.2019.02.002 30743063

[72] Teruel M, Cabrero J, Perfectti F, Camacho JP. 2010. B chromosome ancestry revealed by histone genes in the migratory locust. Chromosoma, 119, 217–225. 20016909 10.1007/s00412-009-0251-3

[73] Traut W, Szczepanowski M, Vítková M, Opitz C, Marec F, Zrzavý J. 2007. The telomere repeat motif of basal Metazoa. Chromosome Research, 15, 371–382. 17385051 10.1007/s10577-007-1132-3

[74] Traldi JB, Ziemniczak K, de Fátima Martinez J, Blanco DR, Lui RL, Schemberger MO, Nogaroto V, Moreira-Filho O, Vicari MR. 2019. Chromosome mapping of H1 and H4 histones in Parodontidae (Actinopterygii: Characiformes): Dispersed and/or co-opted transposable elements? Cytogenetic and Genome Research, 158, 106–113. 31203273 10.1159/000500987

[75] Turčeková L, Hanzelová V, Špakulová M. 2002. Concentration of heavy metals in perch and its endoparasites in the polluted water reservoir in Eastern Slovakia. Helminthologia, 39, 76–80.

[76] Von-Voss H. 1910. Beitrag zur Kenntnis der Eireifung bei den Acanthocephalen. Archiv für Zellforschung, 5, 430–448.

[77] Walton AC. 1959. Some parasites and their chromosomes. Journal of Parasitology, 45, 1–20. 13631567

[78] Yoshido A, Sahara K, Marec F, Matsuda Y. 2011. Step-by-step evolution of neo-sex chromosomes in geographical populations of wild silkmoths, *Samia cynthia* ssp. Heredity, 106, 614–624. 20668432 10.1038/hdy.2010.94 PMC3183898

[79] Yoshido A, Šíchová J, Pospíšilová K, Nguyen P, Šafář J, Provazník J, Voleníková A, Vila R, Marec F. 2020. Evolution of multiple sex-chromosomes associated with dynamic genome reshuffling in *Leptidea* wood-white butterflies. Heredity, 125, 138–154. 32518391 10.1038/s41437-020-0325-9 PMC7426936

[80] Yuuta M, Kazuko KT. 2018. Significance of whole-genome duplications on the emergence of evolutionary novelties. Briefings in Functional Genomics, 17, 329–338. 29579140 10.1093/bfgp/ely007

[81] Zadesentes KS, Katokhin AK, Mordvinov VA, Rubtsov NB. 2012. Comparative cytogenetics of opisthorchid species (Trematoda, Opisthorchiidae). Parasitology International, 61, 87–89. 21798365 10.1016/j.parint.2011.07.006

[82] Zhao TY, Yang RJ, Lü L, Ru SS, Wayland MT, Chen HX, Li YH, Li L. 2023. Phylomitogenomic analyses provided further evidence for the resurrection of the family Pseudoacanthocephalidae (Acanthocephala: Echinorhynchida). Animals, 13, 1256. 37048513 10.3390/ani13071256 PMC10093747

