# Supplementary material for: Chromosome analysis and the occurrence of B chromosomes in fish parasite Acanthocephalus anguillae (Palaeacanthocephala: Echinorhynchida)
Source: Parasite. 2023 Oct 23;30:44. doi: 10.1051/parasite/2023045 (PMC10592040; doi:10.1051/parasite/2023045)
Supplement: Supplementary file 1 — Supplementary Table S1: Summary of karyotype data of acanthocephalan species. [file parasite-30-44-s1.pdf]

Supplementary Table S1 Summary of karyotype data of acanthocephalan species.

**CLASS**<br>ORDER	**Number 2n ♂=X0 / ♀=XX and morphology[a]**	**References[b]**	**Number of 18S rDNA**
**PALAEACANTHOCEPHALA**<br>ECHINORHYNCHIDA			
Acanthocephalus lucii (Müller, 1776)	2n = 7/8, n = 2sm-m + 1sm + 1st(X) + 1-5B<br>2n ♀ = 8, n = 2sm + 1st + 1st(X)	Špakulová et al. 2002 (A)<br>Mutafova et al.1997 (A)	
Acanthocephalus anguillae (Müller, 1780)	2n = 7/8; n = 1m + 1m-sm + 1a + 1a (X) + 1B	present work (B)	three rDNA clusters
Acanthocephalus ranae (Schrank, 1788) (=*Echinorhynchus haeruca* Rudolphi, 1809)	2n = 16	Hamann 1891, reported by Walton 1959	
	2n = 8, n=4m	John 1957 (-)	
Echinorhynchus gadi Zoega in Müller, 1776 (=*Echinorhynchus acus* Rudolphi, 1902)	2n = 16	Hamann 1891, reported by Walton 1959	
Echinorhynchus truttae Schrank, 1788	2n = 8	Parenti et al. 1965	
Leptorhynchoides plagicephalus (Westrumb, 1821)	2n = 7/8, n = 1sm + 1m + 1t + 1t(X)	Fontana et al. 1993a (A)	
Leptorhynchoides thecatus (Linton, 1891)	2n = 5/6, n = 1m + 1sm +1a(X)	Bone 1974a (-)	
Pomphorhynchus laevis (Zoega in Müller, 1776)	2n = 8<br>2n = 7/8, n = 1sm + 1st + 1t + 1t(X)<br>2n = 7/8, n = 2sm + 1st + 1st(X)<br>2n = 7/8, n = 1sm + 1sm + 1a + 1a(X)	Von Voss 1910<br>Mutafova and Nedeva 1988 (A)<br>Fontana et al. 1993b (A)<br>Bombarová et al. 2006 (-)	two rDNA clusters
Pomphorhynchus tereticollis (Rudolphi, 1809)	2n = 7/8, 1sm + 1st + 1a + 1a(X)	Bombarová et al. 2006 (-)	two rDNA clusters
POLYMORPHIDA			
Filicollis anatis (Schrank, 1788) (=*Echinorhynchus polymorphus* Bremser, 1824)	2n = 16	Hamann 1891, reported by Walton 1959	
**ARCHIACANTHOCEPHALA**<br>MONILIFORMIDA			
Moniliformis (Bremser, 1811) (=*Moniliformis dubius* Meyer, 1932)	2n = 7/8, n = 3m + 1m(X)	Robinson 1965 (-)	
OLIGACANTHORHYNCHIDA			
Macracanthorhynchus hirudinaceus (Pallas, 1781)	2n = 6, n = 1sm + 1m + 1st	Jones and Ward 1950 (-)	
Macracanthorhynchus hirudinaceus (Pallas, 1781)	2n = 6, n♀ = 2m + 1a, n♂ = 1m + 1a +1m(X)/1a(Y)	Robinson 1964 (-)	
**EOACANTHOCEPHALA**<br>NEOECHINORHYNCHIDA			
Neoechinorhynchus cylindratus (Van Cleave, 1913)	2n = 5/6, n = 1m + 1sm +1a(X)	Bone 1974b (-)	

[a] Chromosome morphology: a – acrocentric; m – metacentric, sm – submetacentric, st – subtelocentric, t – telocentric chromosome pair, B – supernumerary chromosome, X – sex chromosome.
[b] Chromosome nomenclature according to (A) Levan et al. 1964, (B) Dos Santos Guerra 1986, (-) unspecified.
